# Quantitative methylation profiling in tumor and matched morphologically normal tissues from breast cancer patients

**DOI:** 10.1186/1471-2407-10-97

**Published:** 2010-03-12

**Authors:** Ilse Van der Auwera, Catherine Bovie, Cecilia Svensson, Xuan B Trinh, Ridha Limame, Peter van Dam, Steven J van Laere, Eric A van Marck, Luc Y Dirix, Peter B Vermeulen

**Affiliations:** 1 Translational Cancer Research Group (Laboratory of Pathology, University of Antwerp/University Hospital Antwerp; Oncology Centre, General Hospital St-Augustinus), 2610 Antwerp, Belgium; 2 OncoMethylome Sciences SA, Centre Hospitalier Universitaire, 4000 Liège/Sart Tilman, Belgium

## Abstract

**Background:**

In the present study, we determined the gene hypermethylation profiles of normal tissues adjacent to invasive breast carcinomas and investigated whether these are associated with the gene hypermethylation profiles of the corresponding primary breast tumors.

**Methods:**

A quantitative methylation-specific PCR assay was used to analyze the DNA methylation status of 6 genes (*DAPK, TWIST, HIN-1, RASSF1A, RARβ2 *and *APC*) in 9 normal breast tissue samples from unaffected women and in 56 paired cancerous and normal tissue samples from breast cancer patients.

**Results:**

Normal tissue adjacent to breast cancer displayed statistically significant differences to unrelated normal breast tissues regarding the aberrant methylation of the *RASSF1A *(P = 0.03), *RARβ2 *(P = 0.04) and *APC *(P = 0.04) genes. Although methylation ratios for all genes in normal tissues from cancer patients were significantly lower than in the cancerous tissue from the same patient (P ≤ 0.01), in general, a clear correlation was observed between methylation ratios measured in both tissue types for all genes tested (P < 0.01). When analyzed as a categorical variable, there was a significant concordance between methylation changes in normal tissues and in the corresponding tumor for all genes tested but *RASSF1A*. Notably, in 73% of patients, at least one gene with an identical methylation change in cancerous and normal breast tissues was observed.

**Conclusions:**

Histologically normal breast tissues adjacent to breast tumors frequently exhibit methylation changes in multiple genes. These methylation changes may play a role in the earliest stages of the development of breast neoplasia.

## Background

Breast cancer is the most frequently diagnosed cancer in women worldwide http://www.cancer.org. Approximately 1.3 million women are diagnosed with breast cancer each year. There are well-understood genetic alterations associated with breast carcinogenesis, including specific gene amplifications, deletions, point mutations, chromosome rearrangements and aneuploidy. In addition to these highly characterized mutations, epigenetic alterations are key contributors to breast carcinogenesis. The most widely studied epigenetic event in breast cancer is the hypermethylation of CpG islands associated with the promoter and first exon regions of several genes [1]. Methylation of CpG islands in gene promoter regions is thought to be especially relevant for the silencing of important growth control genes. For breast cancer, some of the genes reported to undergo hypermethylation are involved in evasion of apoptosis (*DAPK, TWIST1, HOXA5*), cell cycle regulation (*p16, CCND2*), cell invasion and metastasis (*CDH1, APC*), DNA repair (*BRCA1*) and cell signaling (*ER *and *RARβ2*) [2]. These epigenetic alterations occur at an early stage in breast carcinogenesis. High levels of some hypermethylated genes can be detected very early, in the ductal lavage and nipple aspirates of patients with ductal carcinoma *in situ *and stage I tumors, with methylation frequencies comparable with those of more advanced, invasive breast cancers [3].

Hitherto, there has been a focus on the biology of the primary tumor and its immediate precursor lesions rather than on the apparently normal epithelial cells in which the carcinogenic sequence begins. Nevertheless, there is a growing realization that the emergence of focal lesions occurs in association with 'field changes', which can be defined as the presence of cancer causing changes in apparently normal tissue surrounding a neoplasm [4]. The presence of field cancerization has been described in different cancer types, including breast cancer [5]. Previous studies have demonstrated that genetic alterations, such as loss of heterozygosity and allelic imbalance, exist in histologically normal breast tissues immediately adjacent to invasive cancers [6,7]. Recently, normal tissue adjacent to primary breast carcinomas has been shown to exhibit hypermethylation changes in multiple genes that are also present in the primary tumor [8-14]. Some of the early epigenetic changes in histologically normal tissues adjacent to e.g. prostate or colon cancer have been shown to be an age-related event [15-18]. However, in breast cancer, the relationship between methylation changes in normal breast tissues from cancer patients and patients' age has not yet been studied in detail.

Quantitative methylation profiling for the identification and classification of field defects might provide an objective approach for early detection or risk assessment of breast cancer. In fact, DNA methylation in benign breast epithelial cells has been related to a personal history of benign or malignant breast disease and to predicted breast cancer risk in two independent studies [19,20]. Promoter methylation of *RASSF1A *showed the greatest discrimination between benign samples from women with breast cancer, unaffected high-risk women and unaffected low-risk women, as defined by the Gail model [19].

In the present study, we used quantitative real-time methylation-specific PCR (qMSP) to quantify the methylation status of 6 genes in matched normal and cancerous tissues from 56 patients with invasive breast cancer: death associated protein kinase (*DAPK*), *TWIST*, high in normal-1 (*HIN-1*), RAS association domain family protein 1A (*RASSF1A*), retinoic acid binding receptor beta 2 (*RARβ2*) and adenomatous polyposis coli (*APC*). The purposes of this study were: i) to measure the frequency of gene hypermethylation in tumor tissue, normal tissue from breast cancer patients and normal tissue from unaffected patients and ii) to determine whether methylation changes in normal tissues from breast cancer patients are associated with age.

## Methods

### Patients and sample collection

We collected 9 normal breast tissue samples from patients who underwent breast reductive surgery (age range, 25-47 years). None of these samples showed pathological changes. In addition, we collected 56 pairs of matched normal and breast cancer tissue samples from patients with breast cancer (age range, 30-86 years). Additional primary tumor characteristics were recorded by review of pathology files and are listed in Table 1. Tumors were histologically graded from 1 to 3 according to the Nottingham modification of the Bloom and Richardson histological grading scheme [21]. ER, PR and P53 status were determined by immunohistochemistry. HER2 status was determined according to the College of American Pathologists (CAP) and the American Society of Clinical Oncology (ASCO) joint guideline [22].

**Table 1:** Patient characteristics

Clinicopathological factors (N = 56)	N (%)
T status	
1	28 (50%)
2	18 (32%)
3	7 (13%)
4	3 (5%)
Nodal involvement	
Negative	30 (54%)
Positive	26 (46%)
American Joint Committee on Cancer Stage	
I	24 (43%)
II	16 (29%)
III	14 (25%)
IV	2 (4%)
Grade	
1	19 (34%)
2	18 (32%)
3	19 (34%)
ER status	
Negative	7 (13%)
Positive	48 (86%)
Unknown	1 (1%)
PR status	
Negative	17 (30%)
Positive	39 (70%)
P53 status	
Negative	40 (71%)
Positive	14 (25%)
Unknown	2 (4%)
HER2 status	
Negative	41 (73%)
Positive	15 (27%)

All samples were procured at the time of surgery, subjected to an initial gross pathological examination, frozen in liquid nitrogen and then stored in N_2 _at -180°C. Corresponding normal tissues were procured at the most distant site from the resected specimen (distances from the primary tumor were not routinely measured in this study). For each tumor and normal breast tissue sample, a section adjacent to the tissue part used for DNA extraction was stained with haematoxylin and eosin for histological confirmation of the presence or absence of cancer cells. However, tissue sizes were inadequate to perform tissue morphometry on these slides.

Informed consent was obtained from all patients participating in the study. All samples were obtained from Sint-Augustinus (Antwerp, Belgium) in accordance with the institutional policies. All protocols were reviewed and approved by the Ethical Committee of Sint-Augustinus.

### Extraction and sodium bisulphite conversion of DNA

DNA extractions from breast tissue samples were performed using the QIAamp DNA Mini Kit (Qiagen, Valencia, CA, USA) according to the manufacturer's protocol. DNA samples (200 μl) were frozen at -80°C until use. DNA was quantified using a NanoDrop 1000 spectrophotometer (Thermo Scientific, Wilmington, DE, USA) and 1.5 μg of DNA was sodium bisulphite-converted using the EZ DNA Methylation Kit (Zymo Research, Orange, CA, USA) according to the manufacturer's instructions.

### Quantitative real-time MSP

The analyte (*RASSF1A, RARβ2, APC, DAPK, HIN1, TWIST1 *and *ACTB*) quantitations were done in real-time PCR assays using the ABI Prism 7900HT (Applied Biosystems, Foster City, CA, USA). Methylated version of *RASSF1A, RARβ2, APC, DAPK, HIN1 *and *TWIST1 *promoter sequences were detected. *ACTB *was used as a reference gene in the assay, using primers that are outside any CpG islands. The PCR conditions were 95°C for 5 min, followed by 45 cycles of 95°C for 30 s, 57°C for 30 s, and 72°C for 30 s, with a final extension cycle of 72°C for 5 min (the annealing temperature was 51°C instead of 57°C for the *APC *assay). Data were collected at the 57°C (or 51°C) plateau.

The results were generated using the SDS 2.2 software (Applied Biosystems, Foster City, CA, USA). The copy numbers were calculated based on the linear regression obtained for a standard curve of 8 to 8 × 10^5 ^gene copy equivalents, using plasmid DNA containing the bisulphite-modified sequence of interest. CpGenome™ Universal methylated and unmethylated DNA (Millipore, Billerica, USA) were included in each experiment as positive and negative controls, respectively.

The amplicons generated during the amplification process were quantified by real-time measurement of the emitted fluorescence (fluorophore: FAM). The ratio between the methylated marker and the independent reference gene *ACTB *was calculated. This ratio was defined as the test result (test result = copies methylated marker/copies *ACTB *× 1,000).

### Statistical analysis

Test results for each gene were analyzed in two ways: as a continuous variable and as a dichotomized variable (according to the maximal methylation ratio observed in normal breast tissues from unaffected women). We used Pearson's Χ2 or, in the case of low frequencies per cell, Fisher's exact method to test associations between categorical variables. The Mann-Whitney U test or the Wilcoxon signed-rank test was used to assess differences between nonparametric distributed variables. The Kappa statistic was used to assess the agreement between two dichotomous variables. A two-sided P ≤ 0.05 was considered to be statistically significant. All statistical calculations were performed using SPSS, version 11.0 (SPSS Inc, Chicago, IL, USA).

## Results

### Gene methylation ratios in matched normal and cancerous breast tissue

A total of 6 genes (*DAPK, TWIST, HIN-1, RASSF1A, RARβ2 *and *APC*) was analyzed for promoter methylation in normal breast tissues from 9 reduction mammoplasty specimens and in matched normal and cancerous tissues from 56 breast cancer patients using qMSP. Results for all genes in all cases are presented in Table 2.

**Table 2:** DNA methylation of 6 genes associated with breast carcinogenesis in normal and cancerous breast tissues.

Gene	Normal tissue from unaffected women (N = 9)	Normal tissue from cancer patients (N = 56)	Cancerous tissue (N = 56)	**P-value**^a^	**P-value**^b^
*DAPK*	0.47 (0.00-2.51)	0.30 (0.00-690.19)	0.92 (0.00-1445.43)	0.56	0.34
*TWIST*	0.00 (0.00-1.78)	0.00 (0.00-388.92)	0.00 (0.00-1066.79)	0.06	0.03
*HIN-1*	0.00 (0.00-0.00)	0.00 (0.00-296.26)	199.46 (0.00-2627.44)	0.31	0.003
*RASSF1A*	0.74 (0.19-103.25)	11.15 (0.00-418.42)	348.80 (0.00-1241.21)	0.03	<0.001
*RARβ2*	0.00 (0.00-0.00)	0.00 (0.00-183.29)	0.00 (0.00-977.59)	0.04	0.07
*APC*	0.00 (0.00-0.00)	0.00 (0.00-1992.07)	163.97 (0.00-4481.91)	0.04	0.004

^a ^Comparison between normal tissues from different sources

^b ^Comparison between normal tissue from unaffected women and cancerous tissue

Median methylation ratios and range are shown.

Some degree of methylation was detectable in normal breast tissue from unaffected women for 3 of the 6 genes assayed (*DAPK, TWIST *and *RASSF1A*), although the ratios of methylation varied considerably for different genes, from a maximal test result of approximately 2 for *DAPK *and *TWIST *to a maximal value of 103 in the case of *RASSF1A*. Compared with the normal breast tissues from unaffected women (N = 9), we observed higher methylation ratios in normal breast tissues from cancer patients (N = 56) for *RASSF1A *(P = 0.03, Mann Whitney test), *RARβ2 *(P = 0.04, Mann Whitney test) and *APC *(P = 0.04, Mann Whitney test). Notably, for the *RASSF1A *gene, the median methylation ratio in normal tissues from cancer patients was 15-fold higher compared with that in normal tissues from unaffected women. For the *DAPK, TWIST *and *HIN-1 *genes, there were no significant differences between the normal tissues from different sources. For all genes, the ratios of methylation in cancerous tissue were higher than in normal breast tissue from unaffected women and for 5 genes (*TWIST, HIN-1, RASSF1A, APC*) these differences were statistically significant (Table 2). The methylation ratios of different genes were not independent of each other. Genes for which methylation ratios most closely correlated in cancerous tissues (N = 56) were *RASSF1A *and *HIN-1 *(r = 0.480, P < 0.001) and *RASSF1A *and *TWIST *(r = 0.438, P = 0.001). Genes for which methylation ratios most correlated in normal tissues from cancer patients (N = 56) were *RARβ2 *and *APC *(r = 0.502, P < 0.001) and *RASSF1A *and *HIN-1 *(r = 0.485, P < 0.001).

Next, we compared the methylation ratios for normal and matched cancerous tissue for each breast cancer patient. For all genes, the ratios of methylation in the cancerous tissue significantly exceeded those of normal tissue from the same patient (P ≤ 0.01, Wicoxon signed-rank test). However, in general, a clear correlation between methylation ratios in normal and cancerous tissues could be detected. Correlation coefficients and corresponding values of significance were: r = 0.378 and P = 0.004 for *DAPK*, r = 0.538 and P < 0.001 for *TWIST*, r = 0.371 and P = 0.005 for *HIN-1*, r = 0.428 and P = 0.001 for *RASSF1A*, and r = 0.491 and P < 0.001 for *APC*.

**Figure 1:**
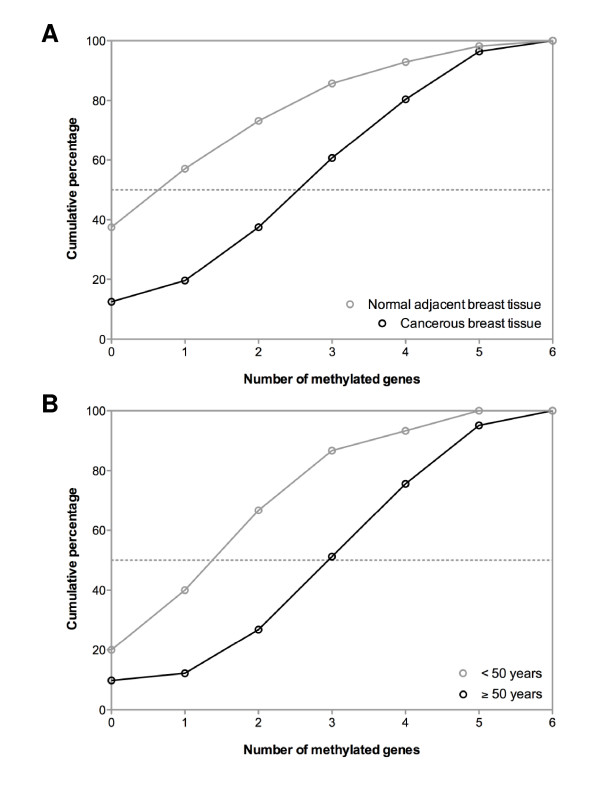
**(A) Cumulative percentage distribution of cancerous (black line) and normal (grey line) samples in function of number of methylated genes**. The median number of hypermethylated genes was 3 in cancerous tissues and 1 in normal breast tissues (P < 0.001); (B) Cumulative percentage distribution of breast cancer tissues from patients ≥50 years (black line) and <50 years (grey line). The median number of hypermethylated genes was 3 in cancerous tissues from patients ≥50 years and 2 in cancerous tissues from patients <50 years (P = 0.006).

As cut-off for scoring a sample as 'hypermethylated', the maximal methylation ratio in the control group (normal breast tissues from unaffected women) was used. The frequency of hypermethylated samples was similar in normal and cancerous breast tissues for *DAPK, TWIST *and *RARβ2*. However, for *HIN-1, RASSF1A *and *APC*, cancerous breast tissues were more frequently hypermethylated than matched normal tissues. The methylation frequencies for cancerous and matched normal tissues were as follows: 27% and 21% for *DAPK *(P Χ^2 ^= 0.51), 46% and 36% for *TWIST *(P Χ^2 ^= 0.25), 59% and 11% for *HIN-1 *(P Χ^2 ^< 0.001), 77% and 18% for *RASSF1A *(P Χ^2 ^< 0.001), 29% and 34% for *RARβ2 *(P Χ^2 ^= 0.54) and 55% and 36% for *APC *(P Χ^2 ^= 0.04). Methylation of at least one of the 6 genes tested was present in 87% of cancerous tissues and 62% of normal tissues (P Χ^2 ^= 0.002). Methylation of multiple genes (three or more genes) was detected in 39% of cancerous tissues compared with 14% of normal tissues (P Χ^2 ^= 0.003). The median number of hypermethylated genes was significantly greater for cancerous tissues than for normal tissues (3 in cancerous tissues and 1 in normal tissues; P < 0.001, Mann Whitney test) (Figure 1A).

### Concordant gene methylation in matched normal and cancerous breast tissue

For most genes, there was a fair to moderate agreement between methylation in cancerous and matched normal tissues as indicated by the Kappa statistics (Table 3). Only *RASSF1A *failed to show statistically significant concordance between the normal tissues and cancerous tissues. Concordant methylation changes in normal and cancerous breast tissues were present in 77% of cases for *DAPK*, in 68% of cases for *TWIST*, in 52% of cases for *HIN-1*, in 37% of cases for *RASSF1A*, in 77% of cases for *RARβ2 *and in 66% of cases for *APC*. When a gene was found hypermethylated in the primary tumor, it was also hypermethylated in the adjacent normal tissue in 47% of cases for *DAPK*, in 54% of cases for *TWIST*, in 18% of cases for *HIN-1*, in 21% of cases for *RASSF1A*, in 69% of cases for *RARβ2 *and in 52% of cases for *APC*. For *DAPK, TWIST, RASSF1A, RARβ2 *and *APC*, in some instances (2-14% of cases), a gene was found hypermethylated in adjacent tissue but not in the corresponding primary tumor. For 41 of 56 (73%) of patients at least one gene with an identical methylation change in cancerous and normal breast tissues was observed.

**Table 3:** Concordance between the methylation status of cancerous and matched normal breast tissues (N = 56).

Gene	T+ N+	T- N+	T- N-	T+ N-	Kappa	P-value
*DAPK*	7 (13%)	5 (9%)	36 (64%)	8 (14%)	0.368	0.005
*TWIST*	14 (25%)	6 (11%)	24 (43%)	12 (21%)	0.344	0.008
*HIN-1*	6 (11%)	0 (0%)	23 (41%)	27 (48%)	0.154	0.03
*RASSF1A*	9 (16%)	1 (2%)	12 (21%)	34 (61%)	0.070	0.27
*RARβ2*	11 (20%)	8 (14%)	32 (57%)	5 (9%)	0.462	<0.001
*APC*	16 (29%)	4 (7%)	21 (37%)	15 (27%)	0.342	0.006

Abbreviations: T, tumor; N, normal

### Association between DNA methylation changes and clinicopathological factors

Next, we investigated whether the presence of hypermethylated genes in normal tissues from cancer patients was associated with clinicopathological features of the corresponding primary tumor. The presence of at least one hypermethylated gene in adjacent normal breast tissues was significantly higher when the corresponding primary tumors were expressing ER (P Χ^2 ^= 0.007) or PR (P Χ^2 ^= 0.03). Furthermore, hypermethylation of *RASSF1A *was more frequently present in adjacent normal breast tissues from advanced stage breast tumors (P Χ^2 ^= 0.01) and hypermethylation of *APC *was more frequently present in adjacent normal breast tissues from breast tumors expressing ER (P Χ^2 ^= 0.04).

### Association between DNA methylation changes and age

Increased DNA methylation in benign breast epithelium has been associated with age [23]. We therefore investigated DNA methylation as a function of age in normal breast tissues from cancer patients and matched breast tumor tissues (N = 56). The mean age of these patients was 58 years (age range, 30-86 years). We did not investigate the association between methylation changes in normal breast tissues from unaffected women and age since the age distribution in this population did not allow for this analysis. For all 6 genes tested, promoter methylation ratios in normal or cancerous breast tissues did not correlate with patients' age.

Next, we compared hypermethylation frequencies in normal and cancerous tissues from women ≥50 years of age (N = 41) and women <50 years of age (N = 15). In normal tissues from cancer patients no differences in hypermethylation frequencies for any of the 6 genes tested were observed between both patient groups. However, in cancerous tissues, hypermethylation frequencies of 2 of 6 genes, *DAPK *(P Χ^2 ^= 0.005) and *HIN-1 *(P Χ^2 ^= 0.003), were significantly higher in patients ≥50 years of age when compared to patients <50 years of age. Furthermore, 49% of cancerous tissues from patients ≥50 years of age showed multiple gene hypermethylation (three or more genes) compared to only 13% of cancerous tissues from patients <50 years of age (P Χ^2 ^= 0.02). Also the median number of hypermethylated genes was significantly higher in cancerous tissues from patients ≥50 years of age: 3 (range, 0-6) versus 2 (range, 0-5) (P = 0.006, Mann Whitney test) (Figure 1B).

## Discussion

Altered DNA methylation is observed in the early stages of breast carcinogenesis. Both atypical hyperplasia and ductal carcinoma *in situ *can be distinguished from normal breast tissues based on gene promoter methylation levels [24,20,30,12]. Hypermethylation of tumor suppressor genes has also been reported in women who are at risk of developing breast cancer but who do not have cancer [23,20]. This abnormal change occurs more frequently in benign breast epithelium of women at high risk for breast cancer than in women at low risk. These findings suggest a possible cancer-predisposing role for DNA methylation.

In the present study, we investigated aberrant methylation of six genes in matched normal and cancerous tissues from 56 patients with breast cancer using a qMSP assay. Genes were selected from the literature for their involvement in breast cancer and have been previously shown to be affected by hypermethylation in breast cancer. We observed no or only low levels of methylation in normal breast tissue samples from unaffected women. Although sample size of normal breast tissues was rather small, also in a previous study analyzing methylation of the *APC *gene promoter in 27 normal breast tissues (obtained from reduction mammoplasty specimens), we observed methylation in only three samples [31]. Despite sampling of the normal tissues at the site most distant from the primary tumor in the resection specimens, normal tissue adjacent to breast cancer displayed statistically significant differences to unrelated normal breast tissues regarding the aberrant methylation of the *RASSF1A, RARβ2 *and *APC *genes. Although methylation ratios in normal tissues from cancer patients were significantly lower than in the cancerous tissue from the same patient, in general, a clear correlation was observed between methylation ratios measured in both tissue types for all genes tested. When analyzed as a categorical variable, there was a statistically significant concordance between methylation changes in normal tissues and in the corresponding tumor for all genes tested but *RASSF1A*. The observed frequencies of gene methylation in the cancerous samples were highly concordant with previous reports http://www.pubmeth.org. Notably, in 73% of patients, at least one gene with an identical methylation change in cancerous and normal breast tissues was observed.

One difficulty in methylation studies is the relative purity of the tissue samples in the cells that may be targets for CpG island methylation. Normal breast tissue samples are largely composed of supportive stromal cells and have actually very little epithelial cells. We confirmed the absence of tumor cells in the normal breast tissues on the control slides but unfortunately tissue sizes were inadequate to perform extensive tissue morphometry. We therefore cannot exclude differences in epithelial content between normal breast tissue samples, nor the contamination by ductal carcinoma *in situ*. Only a handful of other studies have assessed multiple genes in paired cases of cancerous and adjacent normal breast tissues. These studies reported findings similar to ours. Lewis et al. analyzed methylation of 5 genes (*APC, RASSF1A, H-cadherin, RARβ2 *and *CCND2*) using MSP in 17 breast tumors and matched ipsilateral normal breast tissues [20]. Promoter hypermethylation of at least two of these genes occurred most frequently in breast cancer (78% of samples, N = 27) followed by normal tissue from cancer patients (40% of samples, N = 17) and at the lowest frequency in normal tissue from unaffected women recruited from a breast cancer risk assessment clinic (24% of samples, N = 55). For two genes, *RARβ2 *and *APC*, the differences in hypermethylation frequency between normal breast tissues from unaffected women (9% and 26%, respectively), normal breast tissues from cancer patients (32% and 33%, respectively) and cancerous tissues (43% and 57%, respectively) were statistically significant. Consistent with these results, our study and the study by Bovenzi et al. reported *RARβ2 *methylation in, respectively, 34% and 37% of normal tissue samples from cancer patients (N = 8) [8]. Virmani et al. observed a lower frequency (11%) of *APC *hypermethylation in normal tissues from resections for breast cancer (N = 28) [13]. Fackler et al. examined six pairs of cancerous and adjacent tissue from the surgical margins that were histologically normal for methylation of four genes (*RASSF1A, TWIST, cyclin D2 *and *HIN-1*) by qMSP [10]. The cumulative methylation levels of all four genes within adjacent histologically normal breast tissues were significantly lower than in the nearby carcinoma, but significantly higher than those measured in mammoplasty specimens (N = 9). In another study, normal tissue samples from the quadrant opposite of the primary tumor (N = 12) showed methylation of each of the 23 genes examined, except for *CDKN2 *[32]. Using differential methylation hybridization to globally screen CpG islands for methylation alterations in a set of paired cancerous and normal tissues, Yan et al. uncovered a group of loci frequently hypermethylated in normal tissues adjacent to breast tumors [14]. In ~70% of the time, hypermethylation of four of these promoters (*RASSF1A, CYP26A1, KCNAB1 *and *SNCA*) was detected in adjacent tissues whenever these genes were found to be hypermethylated in the primary tumor by qMSP. Furthermore, a careful analysis of *RASSF1A *methylation in normal tissues obtained at a progressively greater distance from the primary tumor suggested a gradient in some but not all of breast samples such that the extent of methylation was greater in the tissue within a 1 cm circumference of the tumor compared with tissue obtained from 2-4 cm.

## Conclusions

In summary, we clearly demonstrate that histologically normal appearing breast tissues from breast cancer patients exhibit frequent aberrant DNA methylation changes that are concordant with the corresponding tumor. This hypermethylation may represent a large field defect of preneoplastic changes that occurs early in carcinogenesis. The fact that the normal breast tissues lack microscopic evidence of malignancy suggests that these changes are not transforming themselves. However, they might permit the future acquisition and accumulation of other genetic and epigenetic changes that do, in time, lead to malignancy. Similar findings have been reported for colon [33,16,34], lung [35-37] and prostate cancer [18]. In colon and prostate cancer, age-related methylation changes have been suggested to contribute to the field defect [15-18]. In our study, no association between epigenetic alterations present in normal breast tissues from cancer patients and patients' age was observed. This observation does not support the hypothesis that the observed promoter hypermethylation of the six genes under investigation starts in the normal breast tissue as a function of age. However, a previous study analyzing benign breast epithelial cell samples obtained by fine-needle aspiration biopsy have related increasing DNA methylation to increasing age [23]. The age-dependent variation in methylation seems to be gene dependent since Bean et al. did not observe an association between IINK4a/ARF promoter methylation in fine-needle aspiration samples from women at high risk for development of breast cancer and patients' age [38].

If methylation changes do indeed occur earlier than abnormal histologically findings and are associated with subsequent development of breast cancer, then methylation markers in breast samples could potentially identify women at increased risk for breast cancer who might be good candidates for targeted screening and prevention strategies. For women diagnosed with breast cancer, it remains to be determined whether the identification of methylated markers in apparently normal tissue adjacent to tumor might be predictive of clinical outcomes, such as local tumor recurrence.

## Competing interests

The authors declare that they have no competing interests.

## Authors' contributions

IVDA: carried out the isolation experiments, performed the statistical analysis, drafted the manuscript. CB and CS: carried out the PCR experiments. RL, XT, PVD, SVL, EVM, PV and LD: conceived of the study and participated in the design and coordination. All authors read and approved the final manuscript.

## Pre-publication history

The pre-publication history for this paper can be accessed here:

http://www.biomedcentral.com/1471-2407/10/97/prepub
